# Integration of physiological and remote sensing traits for improved genomic prediction of wheat yield

**DOI:** 10.1002/tpg2.70110

**Published:** 2025-09-04

**Authors:** Guillermo García‐Barrios, Carlos A. Robles‐Zazueta, Abelardo Montesinos‐López, Osval A. Montesinos‐López, Matthew P. Reynolds, Susanne Dreisigacker, José A. Carrillo‐Salazar, Liana G. Acevedo‐Siaca, Margarita Guerra‐Lugo, Gilberto Thompson, José A. Pecina‐Martínez, José Crossa

**Affiliations:** ^1^ Graduate Program in Genetic Resources and Productivity Colegio de Postgraduados Texcoco, Estado de Mexico Mexico; ^2^ International Maize and Wheat Improvement Center (CIMMYT) Texcoco, Estado de Mexico Mexico; ^3^ Department of Plant Breeding Hochschule Geisenheim University Geisenheim Germany; ^4^ University Center of Exact Sciences and Engineering Universidad de Guadalajara Guadalajara Jalisco Mexico; ^5^ Faculty of Telematics Universidad de Colima Colima Colima Mexico; ^6^ Horticulture and Product Physiology Wageningen University Wageningen The Netherlands; ^7^ Graduate Program in Socioeconomics, Statistics, and Informatics Colegio de Postgraduados Texcoco, Estado de Mexico Mexico

## Abstract

Genomic selection is an extension of marker‐assisted selection by leveraging thousands of molecular markers distributed across the genome to capture the maximum possible proportion of the genetic variance underlying complex traits. In this study, genomic prediction models were developed by integrating phenological, physiological, and high‐throughput phenotyping traits to predict grain yield in bread wheat (*Triticum aestivum* L.) under three environmental conditions: irrigation, drought stress, and terminal heat stress. Model performance was evaluated using both five‐fold cross‐validation and leave‐one‐environment‐out (LOEO) schemes. Under five‐fold cross‐validation, the model incorporating vegetation indices derived from spectral datasets from the grain‐filling phase achieved the highest accuracy. In LOEO validation, the model that included days to heading performed best under irrigation, whereas under drought stress, the model utilizing vegetation indices from the vegetative stage showed the highest accuracy. Under terminal heat stress, three models performed best: one incorporating genotype by environment interaction, one using vegetation indices during the vegetative stage, and one integrating spectral reflectance data from both the vegetative and grain‐filling phases. Although incorporating multiple covariates can improve prediction accuracy or reduce the normalized root mean square error, using an extended model with all available covariates is not recommended due to the marginal predictive accuracy gains, increases in phenotyping, costs and complexity of data collection analysis. Overall, our findings show the importance of tailored phenomic inputs to specific environmental contexts to optimize genomic prediction of wheat yield.

## Core Ideas

- Incorporating high‐throughput phenotyping data as covariates improves the accuracy of genomic prediction models.
- Vegetation indices and PROSAIL‐derived traits measured at grain filling substantially increased accuracy of genomic prediction models.
- Genomic prediction under drought stress was less accurate, highlighting challenges in predicting adverse environments.

## Plain Language Summary

Genomic selection is an approach for improving grain yield in plant breeding by applying statistical models that incorporate genome‐wide marker data. Genomic selection enables the prediction of genetic merit before phenotypic evaluation, thereby reducing the need for extensive field trials. In this study, we examined whether integrating genomic data with phenological, physiological, and remote sensing traits could enhance the accuracy of wheat grain yield predictions. Genomic prediction models were evaluated under three environmental conditions: irrigation, drought, and terminal heat stress. Vegetation indices and information derived from drone data collected after heading substantially improved our model's prediction accuracies. These findings provide practical insights for breeders seeking to optimize trait selection for identifying high‐yielding wheat lines across diverse environments.

**Abbreviations**
ALA: average leaf angle
C_ab_
: chlorophyll a + b content
CI_green_
: green chlorophyll index
CI_red‐edge_
: red‐edge chlorophyll index
DTH: days to heading
F_cover_
: fraction of vegetation cover
FIPAR: fraction of intercepted photosynthetically active radiation
GE: genotype by environment interaction
GEBV: genomic estimated breeding value
HTP: high‐throughput phenotyping
LAI: leaf area index
LAIC_ab_
: leaf area index based on chlorophyll content
LOEO: leave‐one‐environment‐out
MCARI: modified chlorophyll absorption ratio index
NDVI: normalized difference vegetation index
NIR: near‐infrared
NRMSE: normalized root mean square error
SNP: single nucleotide polymorphism
SpRef: spectral reflectance
SR: simple ratio

## 1 INTRODUCTION

One of the hardest challenges for our society this century will be to meet the food demands of a growing population. Meeting this demand sustainably is increasingly complicated by harsher climatic conditions, diminishing water resources for irrigation, shrinking arable land, and increased soil degradation (Senapati et al., 2022).

Genetic improvement offers a sustainable solution to this challenge, as its outcomes are permanent and inheritable. Unlike nutritional and phytosanitary interventions, which require continuous inputs, genetic improvements are passed on from one generation to the next, making them a powerful tool to enhance agricultural sustainability (Acquaah, 2007; Mueller & Van Eenennaam, 2022).

Wheat (*Triticum aestivum* L.) breeders develop new cultivars that meet the expectations of farmers, the industry, and consumers in terms of grain yield, yield stability, agronomic traits (e.g., short stature and lodging resistance), tolerance to biotic stresses (e.g., rusts, smuts, powdery mildew, and fusarium head blight), abiotic stresses (e.g., drought resistance and winter hardiness), as well as nutritional aspects and end‐use quality. For example, semihard to hard wheat is used for bread, soft wheat for confectionery products and cookies, while durum wheat is used for semolina products (e.g., couscous) and pasta (Acquaah, 2007; Fradgley et al., 2024; Reynolds & Braun, 2022).

In wheat breeding programs, methods such as pedigree, bulk, single seed descent, and combinations of these methods have been predominantly used to advance generations and develop new cultivars. There is also a growing interest in leveraging biotechnology and bioinformatics to enhance selection gains (Alahmad et al., 2022).

One adopted approach in wheat breeding is genomic selection, which utilizes multiple single nucleotide polymorphisms (SNPs) to predict individuals’ genetic merit (Meuwissen et al., 2001). In this method, a subset of individuals that have been both genotyped and phenotyped (the training population) is used to train a model that estimates SNP effects. The model then predicts genomic estimated breeding values (GEBVs) for a testing population that has been only genotyped (Crossa et al., 2017; Mueller & Van Eenennaam, 2022; H. Wang, 2023). Genomic selection can increase the rate of gain in a breeding program by increasing selection intensity, selection accuracy, or reducing selection cycle length (Da Silva et al., 2025; Escamilla et al., 2025; F. Wang et al., 2025).

The accuracy of GEBVs is determined by factors such as marker density, training population size, relatedness between training and testing populations, trait heritability, linkage disequilibrium, statistical methods employed, genotype by environment (GE) interactions, nonadditive effects, and the quality of phenotypic data (Bančič et al., 2023; Esposito et al., 2023; Jung et al., 2024; Montesinos‐López et al., 2020; Sandro et al., 2024; Schrauf et al., 2020; Sehgal et al., 2020; Xu et al., 2021).

In this context, high‐throughput phenotyping (HTP) methods have gained attention for their ability to provide detailed and noninvasive data on primary and secondary traits throughout the crop cycle (Alemu et al., 2024; Da Silva et al., 2025; Schrauf et al., 2020). HTP platforms often equipped with RGB, multispectral, hyperspectral, or light detection and ranging (LiDAR) sensors; enable the measurement of canopy architecture, photosynthetic efficiency, and biomass accumulation at varying spatial and temporal scales (Araus & Cairns, 2014). By delivering precise phenotypic information on physiological traits such as radiation use efficiency, biomass accumulation, photosynthesis, and chlorophyll fluorescence estimated at leaf and canopy scales (Robles‐Zazueta et al., 2021), HTP can be used to refine genomic prediction models for complex traits (Crain et al., 2018; van Eeuwijk et al., 2019; Walter et al., 2019).

HTP has been integrated into genomic prediction models through various approaches. For instance, previous studies have employed relationship matrices derived from hyperspectral data in genomic best linear unbiased prediction (GBLUP) to predict grain yield in wheat. This research approach demonstrated that relationship matrices derived from hyperspectral reflectance collected in canopies in the field, and leaf and grains in the lab could effectively predict yield with accuracies comparable to those obtained using marker or pedigree data (Krause et al., 2019; Rincent et al., 2018). Another application of spectral reflectance (SpRef) index data was described by Herr et al. (2024) who used these indices in univariate models as covariates and in multivariate models as secondary response variables to predict grain yield in wheat. Similarly, physiological traits such as canopy temperature, chlorophyll content (SPAD), membrane thermostability, senescence rates (stay‐green), and normalized difference vegetation index (NDVI) have been used in combination with marker data to implement genomic prediction of yield in wheat. The multi‐kernel models integrating physiological and genomic data achieved prediction accuracies 35%–169% higher than models that only used genomic information (Guo et al., 2020).

To complement these statistical approaches, radiative transfer models (RTMs) present a new alternative for breeders to add biological meaning into genomic prediction frameworks as RTMs are mechanistic models that link plant structural and biochemical traits with remote sensing information retrieved from satellites, unmanned aerial vehicles, or point‐based spectroradiometers (Jacquemoud et al., 2009). In particular, the two most widely used RTMs, SAIL (scattering by arbitrary inclined leaves) and PROSPECT (leaf optics model), have been combined to develop the PROSAIL (PROSPECT + SAIL) model. The inversion of the PROSAIL model allows the user to retrieve physiological traits such as leaf chlorophyll, carotenoid and water content, leaf area index (LAI), average leaf angle (ALA) distribution, the fraction of vegetation cover (F_cover_), or the fraction of intercepted photosynthetically active radiation (FIPAR) (Duan et al., 2014; Féret et al., 2017). Phenotyping approaches such as vegetation indices, hyperspectral reflectance relationship matrices, the use of thermal imagery, or the use of RTMs such as PROSAIL in synergy with genomics can help to optimize wheat yield predictions. For these reasons, the objectives of this study were as follows: (i) to evaluate the responses of bread wheat to contrasting environmental conditions (irrigated, drought, and heat stress) using phenological, physiological, and HTP traits and (ii) to compare the predictive performance of genomic prediction models that integrate these traits collected during the vegetative and grain‐filling stages.

**Core Ideas**

- Incorporating high‐throughput phenotyping data as covariates improves the accuracy of genomic prediction models.
- Vegetation indices and PROSAIL‐derived traits measured at grain filling substantially increased accuracy of genomic prediction models.
- Genomic prediction under drought stress was less accurate, highlighting challenges in predicting adverse environments.

## 2 MATERIALS AND METHODS

### 2.1 Study population and field trials

The study population is known as “Molecular Panel” (MOLPAN) which is comprised of 240 bread wheat genotypes selected from a large and diverse genetic pool of approximately 1500 genotypes (pre‐MOLPAN). The selection of MOLPAN lines was done based on its contrasting yield, visual biomass, phenology, and plant height under irrigation and drought stress conditions. Genetic fingerprint information was also taken into consideration to ensure that diversity (e.g., landrace‐derived parents and commercial cultivars) was well‐represented within the panel. The trials were conducted at the Campo Experimental Norman E. Borlaug (CENEB) at International Maize and Wheat Improvement Center (CIMMYT), near Ciudad Obregón, Sonora, Mexico (27°20′ N, 109°54′ W). The panel was grown in three environments: (1) irrigation, (2) drought with only a single irrigation during the crop cycle at the time of sowing, and (3) terminal heat stress achieved through late sowing to expose the plants to high temperatures during the anthesis and grain‐filling stages.

Wheat plants were grown under an α‐lattice experimental design, with two replicates per genotype in each environment (480 plots in total). In the irrigation and terminal heat stress environments, each plot consisted of six rows spaced 20 cm apart, with a plot length of 1.5 m, and drip irrigation. In the drought environment, each genotype was established on two raised beds of 2 m per plot. Sowing took place on November 16 and 30, 2022, for the irrigation and drought environments, respectively, and on March 1, 2023, for the terminal heat environment. The harvest dates were May 12, 2023 (irrigation), April 28, 2023 (drought stress), and June 9, 2023 (terminal heat stress). Grain yield was measured following the methodology described by Pietragalla and Pask (2013) and adjusted to grams per square meter.

### 2.2 Assessment of heading date

Days to heading (DTH) was recorded as the number of days until approximately 50% of the plants or culms in the experimental plot exhibited heading. In this study, heading was defined either as the point when spikes halfway emerged (GS55) or as reaching Zadoks Stage 59, following the criteria established by Zadoks et al. (1974).

### 2.3 Stomatal conductance and chlorophyll fluorescence measurements

Stomatal conductance (*g*_s_) and chlorophyll fluorescence traits were measured using a handheld combined leaf porometer‐fluorometer (LI‐600PF, Li‐COR Biosciences). Measurements were made on the flag leaf during grain filling in the three environments and were conducted from 10:00 a.m. to 1:00 p.m. to minimize the effects from plant circadian rhythm and from measuring during the hottest hours of the day, which can stimulate stomatal closure and affect the measurement of *g*_s_. Two plants were measured per plot with each measurement lasting ∼30 s per leaf. The subsamples were averaged per plot for subsequent data analyses.

From these measurements the quantum yield of photosystem II (Φ_PSII_) was calculated, which estimates the efficiency at which light absorbed by PSII is used to reduce electron acceptor *Q*_A_ and drive photochemistry. The following equation was used:

(1) $$\Phi_{\mathrm{PSII}}=\frac{F_{m^{\prime }}-F_{s}}{F_{m^{\prime }}}$$

where $F_{m^{\prime }}$ is the maximum fluorescence yield in a leaf exposed to actinic light and $F_{s}$ is the steady‐state fluorescence yield of a leaf exposed to actinic light.

For transpiration measurements, the following equation was used with the manufacturer software:

(2) $$\mathrm{Transpiration}=\frac{\mu \left(W_{\mathrm{sam}}-W_{\mathrm{ref}}\right)}{s}$$

where μ is the air flow rate in µmol s^−1^, $W_{\mathrm{sam}}$ is the water vapor content in an air stream interacting with the measured leaf, $W_{\mathrm{ref}}$ is the water vapor content in an air stream before interacting with the leaf both measured as mmol H_2_O mol^−1^ air, and *s* is the leaf area in cm^2^.

### 2.4 Multispectral imaging

High‐resolution aerial images were collected using a RedEdge‐3 multispectral camera (Micasense) mounted on a small airplane flying at an altitude of ∼100 m. The ground sampling distance was ∼6 cm, and the images were georeferenced using ground control points distributed across the experimental station. Image calibration was performed using the white calibration target provided by the manufacturer (Micasense). Images were collected at least twice during the vegetative (from canopy closure to booting) and grain‐filling (from anthesis to milk grain) growth periods in each environment. Subsequently, for each growth period, an average of the spectral bands was calculated.

Image analysis was conducted using photogrammetry software (Pix4D) to stitch images and reconstruct the plots. Reflectance values in the red, green, blue, red‐edge, and near‐infrared (NIR) regions were extracted using ArcGIS (ESRI). These values were used to calculate vegetation indices (Table 1), including NDVI, modified chlorophyll absorption ratio index (MCARI), red‐edge chlorophyll index (CI_red‐edge_), green chlorophyll index (CI_green_), and simple ratio (SR). Furthermore, the inversion of the PROSAIL model was implemented using the *prospect* R package (Féret & de Boissieu, 2024) to simulate traits such as LAI, F_cover_, ALA, FIPAR, chlorophyll a + b content (C_ab_), and LAI based on chlorophyll content (LAIC_ab_) (Jacquemoud et al., 2009).

**TABLE 1:** Vegetation indices and PROSAIL‐derived physiological traits used as covariables to complement genomic prediction models. The formulas for PROSAIL‐derived traits are “NA” as their calculations are based on whole spectral ranges as opposed to specific wavelengths.

Abbreviation	Proxy trait	Equation	Reference
**Vegetation indices**			
CI_green_	Chlorophyll content and LAI	$CI_{\mathrm{green}}=\frac{\rho_{\mathrm{NIR}}}{\rho_{\mathrm{green}}}-1$	Hunt et al. (2011)
NDVI	Biomass, chlorophyll and water content, LAI, response to drought and heat stress, and yield	$\mathrm{NDVI}=\frac{\rho_{\mathrm{NIR}}-\rho_{\mathrm{red}}}{\rho_{\mathrm{NIR}}+\rho_{\mathrm{red}}}$	Jackson & Huete (1991)
MCARI	Chlorophyll content and LAI	$\mathrm{MCARI}=\left[\left(\rho_{700}-\rho_{670}\right)-0.2\;\left(\rho_{700}-\rho_{550}\right)\right]\left(\frac{\rho_{700}}{\rho_{670}}\right)$	Haboudane (2004)
CI_red‐edge_	Chlorophyll content, LAI, and biomass	$CI_{\mathrm{red}-\mathrm{edge}}=\frac{\rho_{\mathrm{NIR}}}{\rho_{\mathrm{red}-\mathrm{edge}}}-1$	Hunt et al. (2011)
SR	Biomass	$\mathrm{SR}=\frac{\rho_{\mathrm{NIR}}}{\rho_{\mathrm{red}}}$	Xue & Su (2017)
**PROSAIL**			
ALA	Average leaf angles in the canopy	NA	Féret & de Boissieu (2024)
C_ab_	Chlorophyll a + b content	NA	Féret & de Boissieu (2024)
FIPAR	Fraction of intercepted photosynthetically active radiation	NA	Féret & de Boissieu (2024)
F_cover_	Fraction of vegetation cover	NA	Féret & de Boissieu (2024)
LAI	Leaf area index	NA	Féret & de Boissieu (2024)
LAIC_ab_	Leaf area index based on chlorophyll content	NA	Féret & de Boissieu (2024)

Abbreviations: *ρ*, reflectance; CI, chlorophyll index; LAI, leaf area index; MCARI, modified chlorophyll absorbance reflectance index; NA, not applicable; NDVI, normalized difference vegetation index; NIR, near‐infrared; SR, simple ratio.

### 2.5 Genotypic analysis

Genotyping by sequencing was performed using the TraitGenetics 25K SNP array, specifically developed for wheat. This array incorporates markers from various sources, including the Affymetrix Axiom platform, the Illumina Infinium Wheat 90 and 660K SNP arrays, and other publicly available wheat SNP resources. The dataset underwent multiple filtering steps, excluding SNPs with more than 50% missing data and a minor allele frequency below 0.05. Missing genotypic data were subsequently imputed using the mean genotype value per marker and after filtering 17,263 SNPs remained.

### 2.6 Statistical analysis

For the traits measured in this study the best linear unbiased estimator (BLUE) was calculated using the R package lme4 (Bates et al., 2015) and implemented through the graphical user interface META‐R version 6.04 (Alvarado et al., 2020) as follows:

(3) Yijrb=μ+Ei+Linej+Repr+IBrepIBRep+εijrb

where $Y_{ijrb}$ is the value of the response variable of the fixed effects of line jth (*i *= 1,…, *I*) in the *i*th environment (*j *= 1,…, *J*) measured in the fixed effects of *r*th replicate (*r *= 1, 2) and the random effect of the IB(Rep)th incomplete block within the *r*th replicate (*b *= 1, 2), assumed to have a mean 0 and variance $\sigma_{\mathrm{IB}(\mathrm{Rep})}^{2}$, μ is the general mean, and *ϵ_ij_* are independent random errors assumed to be a normal variable with mean 0 and variance $\sigma_{ijrb}^{2}$.

In this study, GBLUP models were used to estimate the breeding values of bread wheat for grain yield using two validation approaches to assess the model accuracy, a five‐fold cross‐validation (CV) to predict unseen genotypes within the same environment and a leave‐one‐environment‐out (LOEO) approach to evaluate performance in unknown environments to simulate breeding scenarios in novel environmental conditions. In the following subsections, we describe the 12 main models used in our study:

#### 2.6.1 Models E + G + GE and E + G

One of the statistical GBLUP models evaluated in this study, E + G + GE, assumed that the response variable is modeled as follows:

(4) $$Y_{ij}=\mu +E_{i}+g_{j}+Eg_{ij}+\varepsilon_{ij}$$

where $Y_{ij}$ is the grain yield adjusted value of line jth measured in environment ith, μ is the general mean, $E_{i}$ is the fixed effect of the environment ith(i=1,…,I), $g_{j}\;(j=1,\text{…},J$) is the random effects of lines, $Eg_{ij}$ is the random interaction GE effect, and *ϵ_ij_* are independent random errors assumed to be a normal variable with mean 0 and variance $\sigma^{2}$.

Furthermore, it is assumed that the vector of random effect of lines $g={\left(g_{1},\text{…},g_{J}\right)}^{T}$ is distributed according to a multivariate normal distribution $N_{J}\left(0_{J},\sigma_{g}^{2}G\right)$, while the interaction GE random effects $Eg={\left(Eg_{11},\text{…},Eg_{1J},\text{…},Eg_{21},\text{…},Eg_{IJ}\right)}^{T}$ follow a $N_{IJ}\left(0_{IJ},\sigma_{Eg}^{2}\left(I_{I}⊗G\right)\right)$, with $0_{J}$ (mean vector of g), $0_{IJ}$ (mean vector of Eg), $I_{I}$ and ⊗, the null vector of size J, the null vector of size IJ, the identity matrix of dimensions I×I and the Kronecker product, respectively, and G is the genomic relationship matrix (VanRaden, 2008).

The model (E + G) is the same as the one specified in (4) but without the random interaction effects $Eg_{ij}:$

(5) $$Y_{ij}=\mu +E_{i}+g_{j}+\varepsilon_{ij\;}$$

#### 2.6.2 Models E + G + GE + DTH and E + G + DTH

Another evaluated statistical model, E + G + GE + DTH, was obtained by adding the covariate DTH to model (4).

(6) $$Y_{ij}=\mu +E_{i}+g_{j}+Eg_{ij}+x_{ij}^{\left(\mathrm{DTH}\right)}\beta_{\mathrm{DTH}}+\varepsilon_{ij}$$

where all the components are the same as those defined previously for (4), and $\beta_{\mathrm{DTH}}\;$ is the coefficient effect of the covariate DTH, $x_{ij}^{\left(\mathrm{DTH}\right)}$. The model E + G + DTH is the same as model (6) but without the random interaction $Eg_{ij}$.

#### 2.6.3 Models E + G + GE + Phys and E + G + Phys

The model E + G + GE + Phys is similar to model (6), except that now instead of considering the DTH covariate, it incorporates physiological variables such as *g*_s_, transpiration, and Φ_PSII_.

(7) $$Y_{ij}=\mu +E_{i}+g_{j}+Eg_{ij}+\sum_{l=1}^{3}x_{ijl}^{\left(\mathrm{Phys}\right)}\beta_{l}^{\left(\mathrm{Phys}\right)}+\varepsilon_{ij}$$

where all terms except $\sum_{l=1}^{3}x_{ijl}^{\left(\mathrm{Phys}\right)}\beta_{l}^{\left(\mathrm{Phys}\right)}$ are the same as those defined in model (4), and $x_{ijl}^{\left(\mathrm{Phys}\right)},l=1,2,3$, represents the physiological (Phys) covariates listed earlier and $\beta_{l}^{\left(\mathrm{Phys}\right)},l=1,2,3$ represents its corresponding effects. The model E + G + Phys is the same as model (7) but without the random interaction effects, $Eg_{ij}$.

#### 2.6.4 Models E + G + GE + DerivHTP_VegStage_ and E + G + DerivHTP_VegStage_

These models are like the previous models, but now the vegetation indices and PROSAIL‐derived covariates in the vegetative stages (DerivHTP_VegStage_: CI_red‐edge_, NDVI, LAI_PROSAIL, FCOVER_PROSAIL, ALA_PROSAIL, SR, FIPAR_PROSAIL, CI_green_, C_ab__PROSAIL, LAIC_ab__PROSAIL, and MCARI) are incorporated. Because many vegetation indices share the same spectral features (e.g., reflectance in the green, red, NIR spectra) multicollinearity among covariates should be expected. We retained all vegetation indices in the models to simulate a realistic breeding pipeline in which HTP data are used without variable selection. However, it is recognized that this could affect coefficient stability, therefore future studies should explore dimensionality reduction techniques such as principal component analysis, partial least squares, or variable selection to improve model parsimony. The model E + G + GE + DerivHTP_VegStage_ assumes the following:

(8) $$\begin{array}{ccc} Y_{ij} & = & \mu +E_{i}+g_{j}+Eg_{ij}\\ & & +\sum_{l=1}^{11}x_{ijl}^{\left(\mathrm{DerivHTP}\_\mathrm{VegStage}\right)}\beta_{l}^{\left(\mathrm{DerivHTP}\_\mathrm{VegStage}\right)}+\varepsilon_{ij} \end{array}$$

where, once more, all terms except $\sum_{l=1}^{11}x_{ijl}^{\left(\mathrm{DerivHTP}\_\mathrm{VegStage}\right)}\beta_{l}^{\left(\mathrm{DerivHTP}\_\mathrm{VegStage}\right)}$ are the same as those defined in model (4), and $x_{ijl}^{\left(\mathrm{DerivHTP}\_\mathrm{VegStage}\right)}$ represents the DerivHTP_VegStage_ covariates listed, and $\beta_{l}^{\left(\mathrm{DerivHTP}\_\mathrm{VegState}\right)}$ as their corresponding effects. The model E + G + DerivHTP_VegStage_ is the same as model (8) but without the random interaction effects $Eg_{ij}$.

#### 2.6.5 Models E + G + GE + DerivHTP_GrFill_ and E + G + DerivHTP_GrFill_

Similar to model (6), but now these models incorporate vegetation indices and PROSAIL‐derived traits in grain filling (DerivHTP_GrFill_: CI_red‐edge_, NDVI, LAI_PROSAIL, FCOVER_PROSAIL, ALA_PROSAIL, SR, FIPAR_PROSAIL, CI_green_, C_ab__PROSAIL, LAIC_ab__PROSAIL, and MCARI). The model E + G + EG + DerivHTP_GrFill_ assumes the following:

(9) $$\begin{array}{ccc} Y_{ij} & = & \mu +E_{i}+g_{j}+Eg_{ij}\\ & & +\sum_{l=1}^{11}x_{ijl}^{\left(\mathrm{DerivHTP}\_\mathrm{GrFill}\right)}\beta_{l}^{\left(\mathrm{DerivHTP}\_\mathrm{GrFill}\right)}+\varepsilon_{ij} \end{array}$$

where, once more, all terms except $\sum_{l=1}^{11}x_{ijl}^{\left(\mathrm{DerivHTP}\_\mathrm{GrFill}\right)}\beta_{l}^{\left(\mathrm{DerivHTP}\_\mathrm{GrFill}\right)}$ are the same as those defined in model (4) and $x_{ijl}^{\left(\mathrm{DerivHTP}\_\mathrm{GrFill}\right)}$ represents the DerivHTP_GrFill_ covariates listed, and $\beta_{l}^{\left(\mathrm{DerivHTP}\_\mathrm{GrFill}\right)}$ as their corresponding effects. The model E + G + DerivHTP_GrFill_ is the same as model (9) but without the random interaction effects $Eg_{ij}$.

#### 2.6.6 Models E + G + EG + SpRef and E + G + SpRef

Building upon model (6), these models now incorporate SpRef information (SpRef: R_Blue, R_Green, R_Red, R_RedEdge, and R_NIR). The model E + G + EG + SpRef assumes the following:

(10) $$Y_{ij}=\mu +E_{i}+g_{j}+Eg_{ij}+\sum_{l=1}^{5}x_{ijl}^{\left(\mathrm{SpRef}\right)}\beta_{l}^{\left(\mathrm{SpRef}\right)}+\varepsilon_{ij}$$

where all terms except $\sum_{l=1}^{5}x_{ijl}^{\left(\mathrm{SpRef}\right)}\beta_{l}^{\left(\mathrm{SpRef}\right)}$ are the same as those defined in model (4) and $x_{ijl}^{\left(\mathrm{SpRef}\right)}$ represents the spectral bands, and $\beta_{l}^{\left(\mathrm{SpRef}\right)}$ as their corresponding effects. The model E + G + SpRef is the same as model (10) but without the random interaction effects $Eg_{ij}$.

The 12 models described above constitute the core of this study. To further evaluate their predictive performance, we conducted a systematic analysis by progressively incorporating different groups of variables (Table 2). For example, E + G + DTH was initially tested, then added physiological variables (Phys), resulting in the E + G + DTH + Phys model. Subsequently, vegetation indices from the vegetative stage (DerivHTP_VegStage_) were included, yielding the E + G + DTH + Phys + DerivHTP_VegStage_ model. This was followed by the addition of vegetation indices collected during the grain‐filling stage (DerivHTP_GrFill_), leading to the comprehensive E + G + DTH + Phys + DerivHTP_VegStage_ + DerivHTP_GrFill_ model. This stepwise approach enabled the evaluation of all possible variable combinations, ultimately resulting in 40 additional models, 52 possible combinations in total.

**TABLE 2:** Performance of models that consider only one group of variables under a five‐fold cross‐validation scheme.

Model	Accuracy	NRMSE
E + G	0.57	0.149
E + G + DTH	0.57	0.150
E + G + Phys	0.58	0.148
E + G + DerivHTP_VegStage_	0.62	0.131
E + G + DerivHTP_GrFill_	0.71	0.120
E + G + SpRef	0.68	0.115
E + G + GE	0.56	0.121
E + G + GE + DTH	0.57	0.122
E + G + GE + Phys	0.55	0.121
E + G + GE + DerivHTP_VegStage_	0.61	0.116
E + G + GE + DerivHTP_GrFill_	0.70	0.111
E + G + GE + SpRef	0.68	0.105

Abbreviations: DerivHTP_GrFill_, vegetation indices and PROSAIL‐derived traits in grain filling; DerivHTP_VegStage_, vegetation indices and PROSAIL‐derived traits in vegetative stage; DTH, days to heading; E, environment; G, genotype; GE, genotype by environment interaction; NRMSE, normalized root mean square error; Phys, physiological variables (stomatal conductance, transpiration, and Φ_PSII_); SpRef, spectral reflectance.

The Bayesian estimation of all GBLUP models was performed using the BGLR R package (Pérez & De Los Campos, 2014), using 20,000 iterations, with the first 5000 discarded as burn‐in, and a default thinning of five. Flat prior distributions were used for the environment effects ($E_{i}$) and the effect of the covariate DTH ($\beta_{\mathrm{DTH}}$); these were specified using model = “FIXED” in BGLR, which treats these predictors as fixed (nonrandom) effects. For the remaining fixed effects ($\beta_{l}^{\left(\mathrm{Phys}\right)},l=1,2,3;\;\beta_{l}^{\left(\mathrm{DerivHTP}\_\mathrm{VegStage}\right)},i=1,\text{…},11;\beta_{l}^{\left(\mathrm{DerivHT}P_{\mathrm{GrFill}}\right)},i=1,\text{…},11;\beta_{l}^{\left(\mathrm{SpRef}\right)},i=1,\text{…},5),$ independent normal distributions with mean 0 and $\sigma_{g}^{2}$ were assigned for each group of covariates $g\in \{\mathrm{Phys},\mathrm{DerivHT}P_{\mathrm{vegState}},\mathrm{DerivHT}P_{\mathrm{GrFill}},\;\mathrm{SpRef}\}$. These priors correspond to a Bayesian ridge regression (BRR) specification in BGLR (model = BRR). Additionally, a scaled inverse chi‐squared distribution was used for each $\sigma_{g}^{2}$.

The five‐fold CV scheme involved dividing the dataset into training and testing sets. In each iteration, the models were trained using four of the five folds (80% of the data) and evaluated on the remaining fold (20%), ensuring that individuals in the training set were not included in the testing set. This process was repeated five times, with each fold serving as the testing set once.

In each CV fold, the normalized root mean square error (NRMSE) and prediction accuracy were evaluated. Prediction accuracy was defined as the Pearson correlation coefficient between the predicted breeding values and observed phenotypes.

These same 40 models were also used in the evaluation of their prediction accuracy under the LOEO validation using phenotypic information from two environments to predict a third (e.g., Ortiz et al., 2023). Graphical visualization of the results was performed using the R packages reshape2 (Wickham, 2007) and ggplot2 (Gómez‐Rubio, 2017).

## 3 RESULTS

### 3.1 Agronomic, phenological, and physiological traits

**FIGURE 1:**
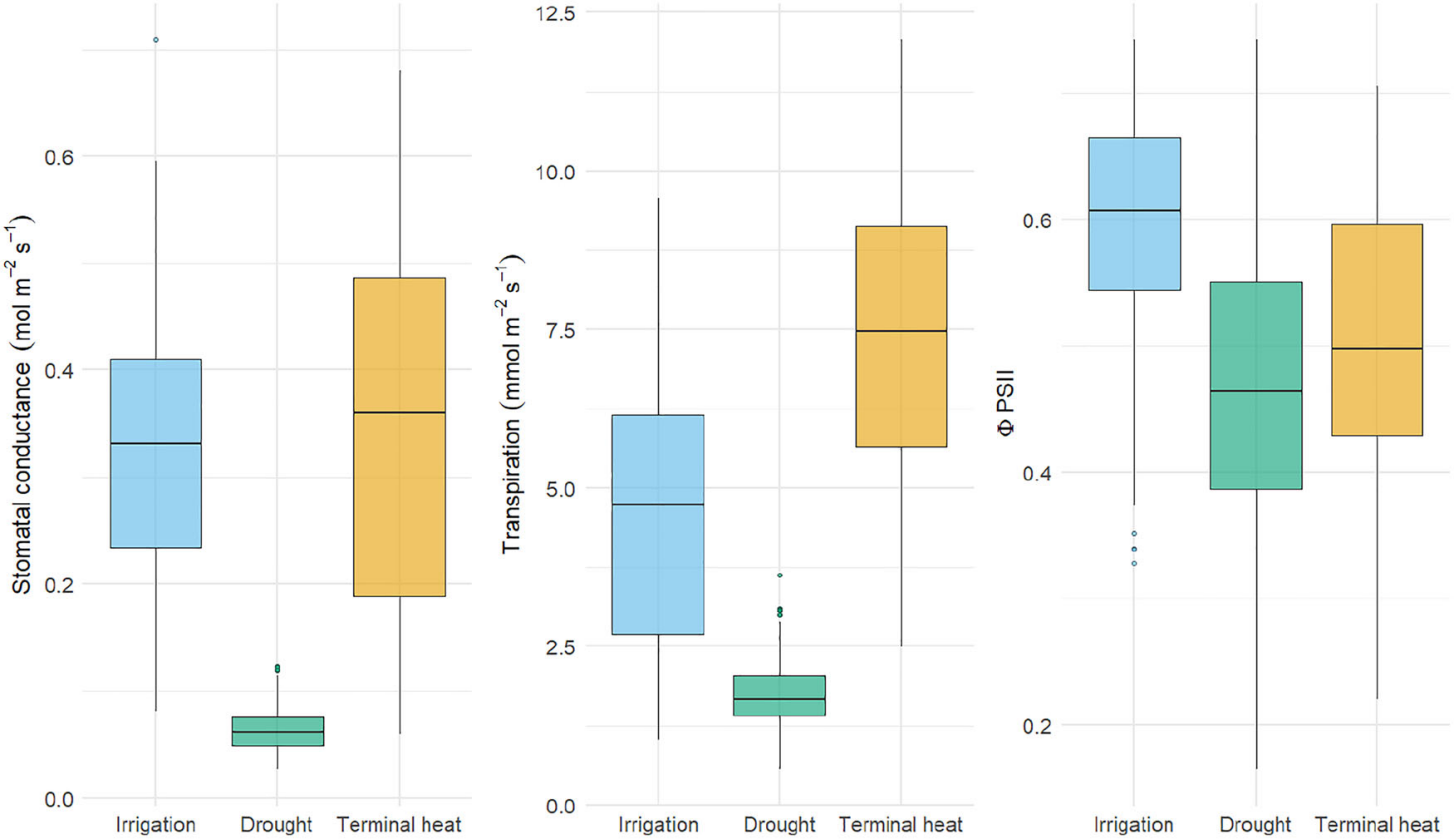
Distribution of stomatal conductance (*g*_s_), transpiration, and quantum yield (Φ_PSII_) measured at grain filling across three environments. Blue, green, and yellow boxplots represent irrigation, drought, and terminal heat environments, respectively.

The MOLPAN bread wheat population was evaluated under three environments: irrigation, drought stress, and terminal heat stress. Under irrigation, the average grain yield was 898.48 g m^−2^, with values ranging from 204.5 to 1148 g m^−2^. In drought stress, the average yield decreased to 396.69 g m^−2^, ranging from 239.1 to 494.91 g m^−2^ and in the terminal heat stress environment, mean yield was 511.65 g m^−2^, ranging from 229 to 665 g m^−2^ (Table S1). Associations between environments showed a high correlation between irrigation and terminal heat stress and low associations of these two environments with drought (Figure S1). Under irrigation, heading occurred at an average of 84 days, dropping to 74 days under drought, and 52 days under terminal heat stress (Table S1). Stomatal conductance (*g*_s_) had a mean of 0.33, 0.06, and 0.34 mol m^−^^2^ s^−1^ in irrigation, drought, and terminal heat conditions, respectively (Figure 1).

Transpiration rates were the lowest at drought environment (1.73 mmol m^−2^ s^−1^), followed by the irrigation environment (4.48 mmol m^−2^ s^−1^) and the highest rates were measured at terminal heat stress (7.38 mmol m^−2^ s^−1^). Φ_PSII_ had an average of 0.59 under irrigation, 0.47 under drought, and 0.50 under heat stress environments, respectively (Figure 1; Table S1).

### 3.2 High‐throughput traits

**FIGURE 2:**
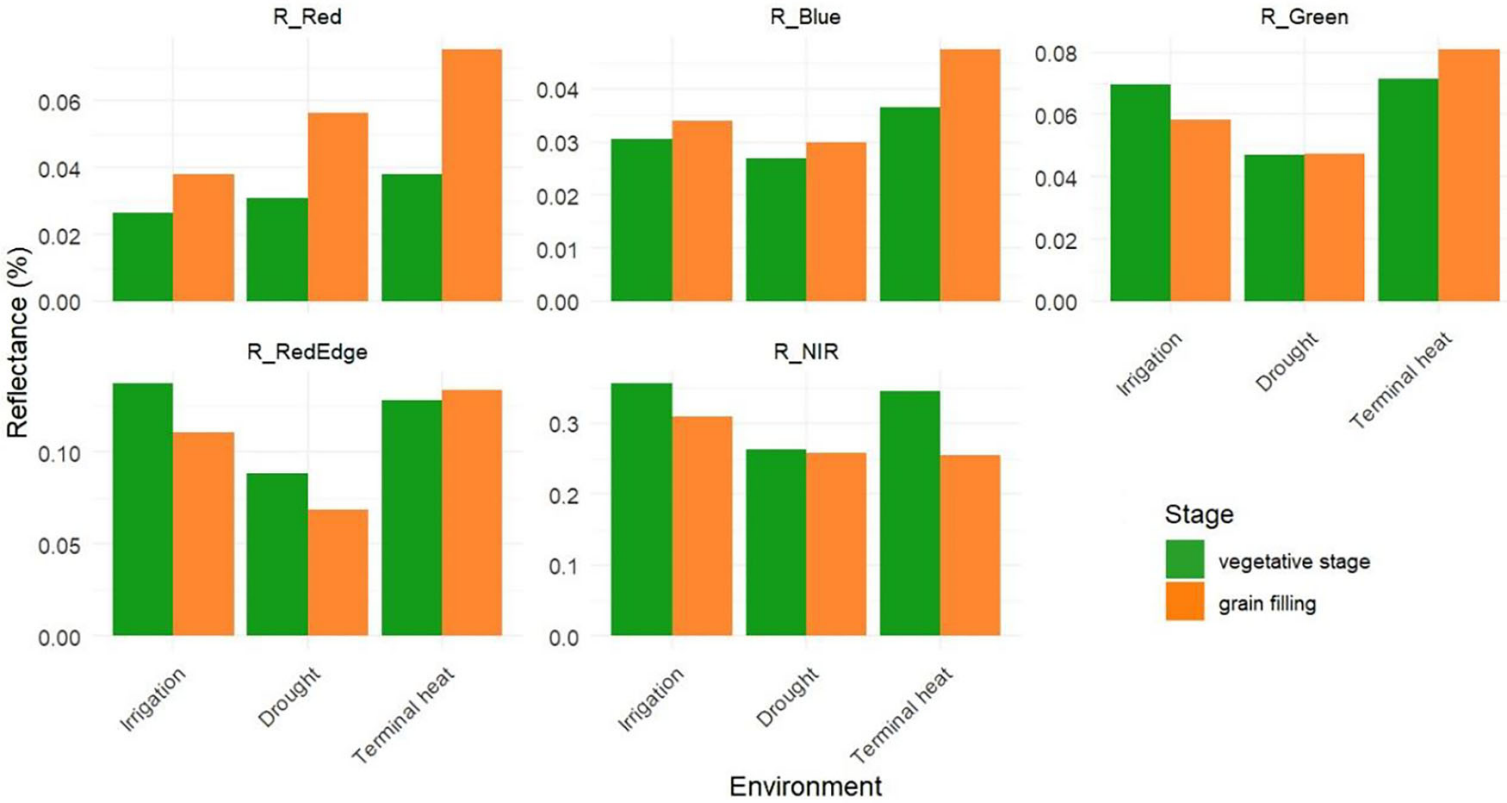
Mean reflectance values (%) measured in five spectral bands; red (R_Red), blue (R_Blue), green (R_Green), red edge (R_RedEdge), and near‐infrared (R_NIR), for wheat grown under three environments: optimal irrigation, drought, and terminal heat. Green and orange bars represent traits measured at the vegetative and grain filling stages, respectively.

Five spectral bands were measured in all environments at the vegetative and grain‐filling stages. In general, reflectance values in the green, NIR, and red‐edge bands were higher at the vegetative phase compared to grain‐filling, in contrast, reflectance in the red and blue band showed higher values during the grain‐filling stage (Figure 2).

**FIGURE 3:**
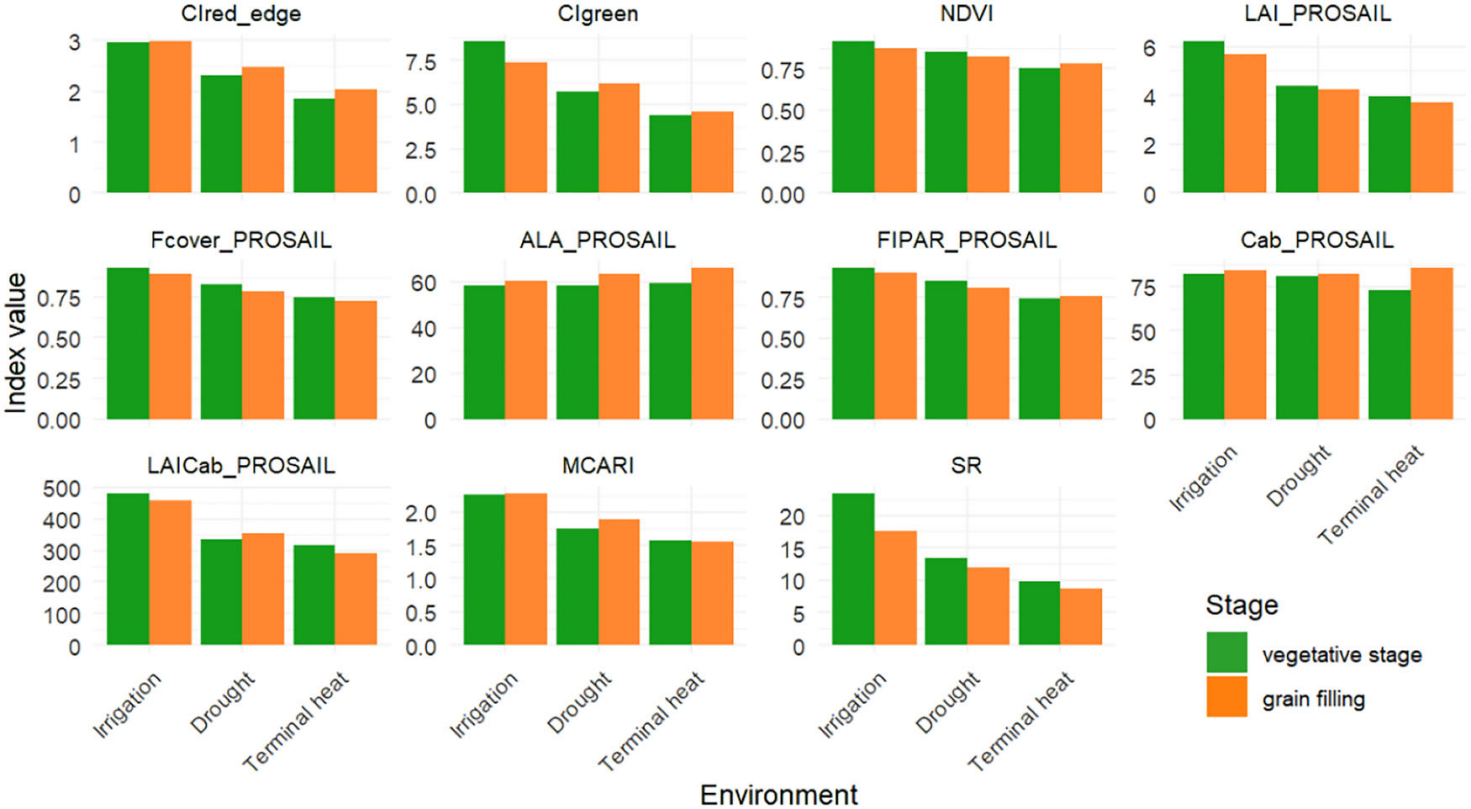
Vegetation indices in three environments (optimal irrigation, drought, and terminal heat), green and orange bars represent traits measured at the vegetative and grain‐filling stages, respectively. ALA, average leaf angle; C_ab_, chlorophyll a + b content; CI_green_, green chlorophyll index; CI_red‐edge_, red‐edge chlorophyll index; F_cover_, fraction of vegetation cover; FIPAR, fraction of intercepted photosynthetically active radiation; LAI, leaf area index; LAIC_ab_, leaf area index based on chlorophyll content; MCARI, modified chlorophyll absorbance reflectance index; NDVI, normalized differenced vegetation index; SR, simple ratio.

Under irrigation conditions, values for CI_red‐edge_, NDVI, LAI_PROSAIL, F_cover__PROSAIL, SR, FIPAR_PROSAIL, CI_green_, LAIC_ab__PROSAIL, and MCARI were the highest among the three evaluated environments (Figure 3).

During the vegetative phase, LAI_PROSAIL, F_cover__PROSAIL, SR, and FIPAR_PROSAIL reached their highest values compared to the grain‐filling stage across all environments. In contrast, ALA_PROSAIL and C_ab__PROSAIL exhibited higher values during the grain‐filling phase (Figure 3).

### 3.3 Evaluating genomic prediction accuracy from five‐fold CV

In the five‐fold CV, among the models that included only a single group of covariates, the best‐performing was E + G + DerivHTP_GrFill_, with a prediction accuracy of 0.71 and an NRMSE of 0.12. In contrast, the worst‐performing model was E + G + DTH, with a prediction accuracy of 0.57 and an NRMSE of 0.15. Within this set of models, the most accurate model (E + G + DerivHTP_GrFill_) outperformed the least accurate model (E + G + DTH) by a margin of 24.5% in accuracy (Table 2).

Among the models that included combinations of variables, three achieved a prediction accuracy of 0.75: E + G + DTH + DerivHTP_GrFill_ + SpRef, E + G + GE + DTH + Phys + DerivHTP_GrFill_, and E + G + GE + DTH + DerivHTP_GrFill_ + SpRef. From these, the last model performed the best in terms of NRMSE (0.099). Additionally, models incorporating vegetation indices from the grain‐filling stage outperformed those using indices from the vegetative phase by 10.9% (Table S2).

### 3.4 Evaluating genomic prediction accuracy using LOEO validation

**FIGURE 4:**
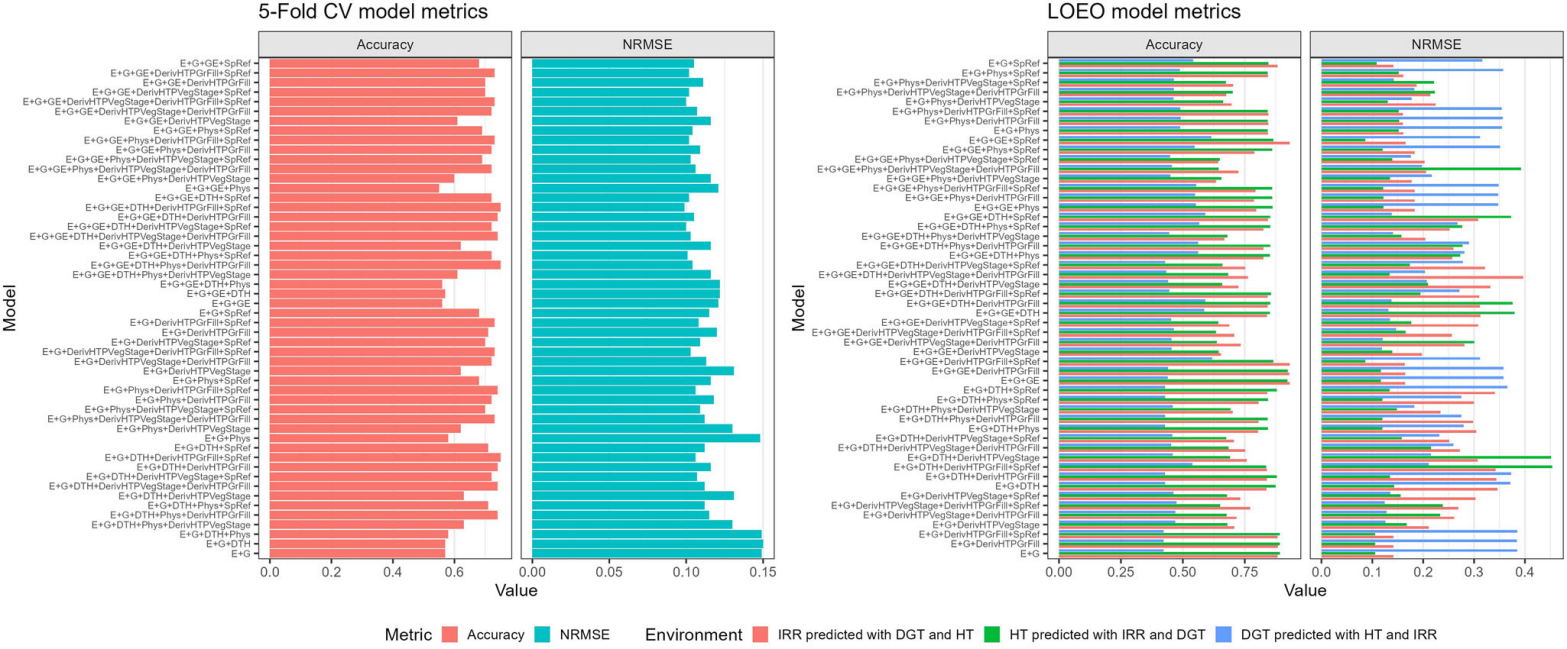
Model metrics for the five‐fold cross‐validation (CV) (left panel) and leave‐one‐environment‐out (LOEO; right panel) schemes. Red bars represent the model accuracy, cyan bars the normalized root mean square error (NRMSE). For the right panel, orange represents the irrigated (IRR) environment, green represents the terminal heat stress (HT) environment, and blue represents the drought (DGT) environment.

In the LOEO validation, phenotypic data from two environments (training) were used to predict grain yield in a third, unobserved environment (validation). For example, grain yield data from irrigation and drought stress environments were used to predict yield under terminal heat stress. The results showed differences in prediction accuracy across the evaluated environments. Across the 52 models tested, the average prediction accuracy in the irrigation environment was 0.78, with an NRMSE of 0.52 (Figure 4; Table S3). The lowest prediction accuracy was observed in the drought stress environment, with an average accuracy of 0.50 and an NRMSE of 0.42. In contrast, under terminal heat stress, the average prediction accuracy was 0.76, with an NRMSE of 1.01 (Figure 4; Table S3).

Under irrigated conditions, all models achieved high prediction accuracy. Among the models that included only a single group of covariates, E + G + DTH and E + G + GE + DTH performed best, each reaching an accuracy of 0.82 (Table 3). The highest prediction accuracy among models incorporating multiple covariate groups was 0.83, observed in both E + G + GE + DTH + Phys + DerivHTP_VegStage_ and E + G + GE + DTH + DerivHTP_VegStage_ + DerivHTP_GrFill_ (Table S3). However, these extended models outperformed the best single covariate model by only 1.2%, indicating a marginal improvement. Overall, models including the DTH variable consistently showed strong predictive performance (Table S3).

**TABLE 3:** Performance of models that consider only one group of variables using the leave‐one‐environment‐out (LOEO) validation approach.

	Irrigation		Drought stress		Terminal heat stress	
Model	Accuracy	NRMSE	Accuracy	NRMSE	Accuracy	NRMSE
E + G	0.79	0.465	0.42	0.385	0.76	0.764
E + G + DTH	0.82	0.515	0.42	0.371	0.75	1.309
E + G + Phys	0.75	0.450	0.42	0.499	0.76	0.640
E + G + DerivHTP_VegStage_	0.73	0.571	0.57	0.470	0.77	1.027
E + G + DerivHTP_GrFill_	0.79	0.465	0.42	0.384	0.76	0.764
E + G + SpRef	0.79	0.465	0.42	0.384	0.76	0.763
E + G + GE	0.80	0.468	0.43	0.358	0.79	0.764
E + G + GE + DTH	0.82	0.549	0.44	0.277	0.74	1.221
E + G + GE + Phys	0.74	0.454	0.44	0.455	0.72	0.649
E + G + GE + DerivHTP_VegStage_	0.79	0.578	0.57	0.393	0.74	0.975
E + G + GE + DerivHTP_GrFill_	0.79	0.468	0.44	0.357	0.79	0.763
E + G + GE + SpRef	0.80	0.468	0.44	0.357	0.79	0.763

Abbreviations: DerivHTP_GrFill_, vegetation indices and PROSAIL‐derived traits in grain filling; DerivHTP_VegStage_, vegetation indices and PROSAIL‐derived traits in vegetative stage; DTH, days to heading; E, environment; G, genotype; GE, genotype by environment interaction; NRMSE, normalized root mean square error; Phys, physiological variables (stomatal conductance, transpiration, and Φ_PSII_); SpRef, spectral reflectance.

In the drought stress environment, among the models that included only a single group of variables, the best‐performing model was E + G + GE + DerivHTP_VegStage_, achieving a prediction accuracy of 0.57 and an NRMSE of 0.39 (Table 3). Among the models incorporating multiple variable combinations, the highest accuracy was observed in E + G + DTH + Phys + DerivHTP_VegStage_, with a prediction accuracy of 0.62 and an NRMSE of 0.245. Notably, in this environment, models integrating vegetation indices from the vegetative phase exhibited strong predictive performance (Table S3).

Under terminal heat stress, among the models with a single covariate group, the best performing were E + G + GE, E + G + GE + DerivHTP_GrFill_, and E + G + GE + SpRef, each achieving a prediction accuracy of 0.79 (Table 3). Among the models incorporating multiple variable combinations, the highest accuracy (0.80) was observed in E + G + DTH + DerivHTP_VegStage_ and E + G + DerivHTP_VegStage_ + DerivHTP_GrFill_ + SpRef, with NRMSE values of 1.245 and 1.169, respectively (Table S3). Notably, in this environment, models that included physiological covariates exhibited the lowest NRMSE values (Table S3).

## 4 DISCUSSION

### 4.1 Effects of stress conditions on physiological traits and canopy reflectance

In this study, the inclusion of biologically relevant covariates can be seen as a pathway to guide breeders to better genomic prediction results. For example, if a trait such as DTH is known to affect yield formation and source‐sink balance, including it in the models helps reducing the environmental noise and improve the genetic values prediction. Similarly, remote sensing datasets that include NDVI‐ or PROSAIL‐derived traits can capture light interception, canopy development, and biomass accumulation dynamics over the growth cycle providing proxies of yield potential.

Late heat stress in cultivars led to higher reflectance values in the visible region (400–700 nm), likely due to reduced chlorophyll content and decreased light absorption (Sareen et al., 2024). This reduction might explain why chlorophyll‐related vegetation indices (CI_red‐edge_, CI_green_, LAIC_ab_, and MCARI) were lower under terminal heat, higher under drought, and peaked with irrigation (Figure 3).

Stomatal conductance (*g*_s_), which measures the rate of CO_2_ into the leaf and water vapor out of the leaf through the stomata, decreases under drought conditions due to partial stomatal closure. This physiological mechanism reduces water loss through transpiration but also limits CO_2_ assimilation and the passive uptake and transport of nutrients (Flexas & Medrano, 2002; Liu et al., 2025). In the present study, comparable values of *g*_s_ were observed under irrigation and terminal heat stress (Figure 1). However, under drought stress, the average *g*_s_ was 0.06 mol m^−^^2^ s^−1^, highlighting the plants’ response to reduced stomatal aperture and the canopy strategy to conserve water under stress conditions.

In certain scenarios, drought avoidance is accomplished by reducing the water‐use rate, thereby extending the time required to deplete soil reserves. This approach is associated with transpiration efficiency, as higher efficiency results in greater biomass production with limited water supply (Ribaut, 2006). In this study, the average transpiration rates were 4.48, 1.73, and 7.38 mmol m^−^^2^ s^−1^ under irrigation, drought, and terminal heat conditions, respectively. However, no consistent correlation was found between transpiration and grain yield across environments. Although a weak but statistically significant association (*p* < 0.05) was observed under terminal heat (*r* = 0.16; Figure S2), this minimal relationship suggests that transpiration rates were not a major determinant of yield in this study.

The lack of correlation between transpiration and yield could be attributed to several factors. First, transpiration rates were measured at a specific growth stage, which may not represent the behavior throughout the entire growing season. Second, soil water availability may have influenced the results, as different soil types have varying water retention capacities. A soil with high retention capacity could have provided a more consistent water supply, thereby masking its relationship with yield (Andrade et al., 2009; Ribaut, 2006).

In this study, Φ_PSII_ values were 0.59 under irrigation, 0.47 under drought, and 0.50 under terminal heat conditions. These results support the hypothesis that stress conditions in plants induce a reduction in electron transfer activity, leading to a decrease in photochemical efficiency and an increase in regulated heat dissipation (Haque et al., 2014; Liu et al., 2025; Luo et al., 2022; Molina‐Salazar et al., 2024).

### 4.2 Prediction accuracy in the five‐fold CV scheme

In our models, we have assumed additive linear effects for covariates for simplicity and model parsimony, however we acknowledge that in future studies nonlinear modeling frameworks such as splines, kernels, or deep learning should be tested to capture more complex trait‐response relationships. In the five‐fold CV scheme, three models incorporating combinations of variables achieved a prediction accuracy of 0.75. These models outperformed the best model with a single covariate group by 5.6% in accuracy. Notably, all four models utilized vegetation index data collected during the grain‐filling stage.

These results are consistent with previous findings by Guo et al. (2020) and Krause et al. (2019), who reported that models incorporating information collected in late‐season growth stages, such as heading and grain‐filling, exhibited higher prediction accuracy compared to models based on vegetative phase.

The three best models also included the DTH covariate. Regarding this variable, it has been established that late‐maturing genotypes tend to have higher yields compared to early maturing ones, as they benefit from a longer period to accumulate biomass (Ibrahim et al., 2024). However, some researchers have pointed out that maturity may introduce confounding effects in yield estimates.

Guo et al. (2020) confirm that correcting grain yield for DTH prevents prediction from being influenced by maturity differences among lines, which is particularly important when evaluating physiological traits that are correlated with phenology. In soybean breeding programs, yield phenotypes are often adjusted to more accurately estimate the intrinsic irrigation, avoiding indirect selection for late maturity (Escamilla et al., 2023; Moreira et al., 2020). Moreno‐Amores et al. (2020) addressed the challenge posed by negative correlations between Fusarium head blight resistance and agronomic traits such DTH and plant height in durum wheat. To facilitate effective selection, they corrected Fusarium severity phenotypes through linear regressions using DTH and plant height as covariates. Additionally, they proposed the use of restriction indices to adjust genomic predictions, offering a strategy to better manage unfavorable trait correlations.

Including GE interaction in our prediction models did not increase the Pearson correlations between observed and predicted phenotypes. However, in all cases, it reduced the NRMSE. These results are aligned with previous reports of increased prediction stability by incorporation of GE interactions (Montesinos‐López et al., 2022; Ortiz et al., 2023; Song et al., 2024).

### 4.3 Prediction accuracy in the LOEO validation scheme

The LOEO validation scheme was used to assess whether the models are robust and capable of predicting performance in new environments (not observed during training).

The best‐predicted environment was irrigation, in contrast, the worst predicted environment was drought. Our results showed that irrigated and heat environment were closely associated (*r* = 0.792) as water is not a limiting factor, in contrast the drought environment has smaller yields and therefore lower correlation to irrigated environments (both optimal and terminal heat stress irrigation) (Figure S1). No model consistently performed well across all three environments. For instance, the E + G + SpRef model performed well under irrigation, achieving a strong Pearson correlation (0.79) and a low NRMSE (0.465) (Table S3). However, this same model showed considerably lower performance under drought conditions, with a Pearson correlation of only 0.42 and an NRMSE of 0.384 (Table S3). This could be attributed to differences in GE interaction and the specific characteristics of each environment.

It has been reported that GE interactions vary significantly across environments (McBreen et al., 2024; Sørensen et al., 2023) and are influenced by both the specific environmental conditions and the genetic responses of the genotypes. Additionally, in many cases, GE interaction patterns are complex and nonlinear, posing a considerable challenge for traditional genomic prediction models.

**FIGURE 5:**
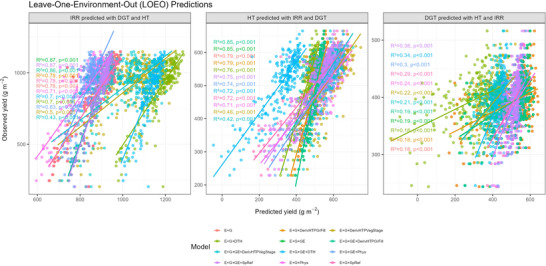
Scatterplot of observed versus predicted grain yield in the leave‐one‐environment‐out (LOEO) scheme with the 12 models that consider only one group of variables. The left panel is the irrigated (IRR) environment predicted with the drought (SQ) and terminal heat stress (HT) environments, the middle panel is the terminal heat stress (HT) environment predicted with the irrigated (IRR) and drought (DGT) environments, and the left panel is the drought (DGT) environment predicted with the terminal heat stress (HT) and irrigated (IRR) environments.

In the LOEO CV scheme, evaluating the impact of covariates can be problematic because this scheme focuses on extrapolation to unobserved environments that are omitted from the training set. In our particular case, this meant predicting terminal heat stress with the irrigation environment, irrigation with the drought environment, and drought with the terminal heat stress environment (Figure 5). This makes it challenging to determine how covariates (phenological, SpRef, vegetation indices, and physiological variables) contribute to improving prediction in other environments, especially when the conditions of unobserved sites fall outside the range of conditions in the sites used for training (McBreen et al., 2024).

For example, grain yield values across environments ranged from 204.5 to 1148 g m^−2^ under irrigation, 239.1 to 494.91 g m^−2^ under drought, and 229 to 665 g m^−2^ under terminal heat stress (Figure 5; Figure S3); similarly, transpiration rates under irrigation ranged from 1.03 to 9.56 mmol m^−^^2^ s^−1^; under drought, from 0.56 to 3.63 mmol m^−^^2^ s^−1^; and under terminal heat, from 2.49 to 12.08 mmol m^−^^2^ s^−1^. This demonstrates that the test environments may present yield, physiological, or remote sensing trait values not represented in the training set, potentially compromising the model's ability to make accurate predictions in these scenarios as showcased by the yield predictions under drought (Figure 5).

### 4.4 Covariates that increase prediction accuracy

For ease of the discussion in this section, results from the LOEO validation scheme were excluded for the reasons outlined in Section 4.3.

If a breeding program could integrate only one group of covariates, the best option would be vegetation indices and PROSAIL traits derived from spectral datasets collected during the grain‐filling stage, as demonstrated by the performance of both the E + G + DerivHTP_GrFill_ model and its equivalent E + G + GE + DerivHTP_GrFill_, which includes GE interactions. Prediction accuracy can be further improved by combining multiple variables. For example, the models E + G + DTH + DerivHTP_GrFill_ + SpRef, E + G + GE + DTH + DerivHTP_GrFill_ + SpRef, and E + G + GE + DTH + Phys + DerivHTP_GrFill_ achieving a prediction accuracy of 0.75 and NRMSE values ranging from 0.099 to 0.106.

However, since models incorporating multiple groups of variables outperformed the best model with a single covariate group (E + G + DerivHTP_GrFill_) by only 5.6%, breeders must determine whether this marginal improvement in prediction accuracy justifies the additional costs, time, and complexity required for data collection, processing, and analysis pipelines.

Finally, to optimize the selection process, it is recommended that breeders use a selection index for the simultaneous improvement of multiple traits. This approach can help balance selection pressure across different traits, minimizing the risk of indirect selection for undesirable traits, such as late maturity.

## 5 CONCLUSIONS

Our results showed that under drought, there was a significant decline in *g*_s_ and Φ_PSII_ rates; however, the terminal heat environment had comparable rates to the irrigated one, confirming that water limitation, rather than heat stress per se, was the primary driver of physiological constraints under abiotic stress.

The models that combined vegetation indices with PROSAIL‐derived traits measured at grain filling achieved the highest yield prediction accuracy. This emphasizes the importance of targeting relevant phenological stages for data collection to improve the model prediction accuracies. Moreover, the consistency of certain covariates such as DTH or LAI (in combination with other traits) to be important across environments suggests that a core set of traits could be routinely phenotyped in breeding programs with low‐cost methods or HTP tools.

While models trained within a certain environment can perform well under similar environmental conditions, they often fail to generalize across novel environments. Therefore, future studies should prioritize modeling frameworks such as nonlinear algorithms or multi‐environment calibration to better capture GE interactions.

## AUTHOR CONTRIBUTIONS

**Guillermo García‐Barrios**: Formal analysis; investigation; methodology; visualization; writing—original draft; writing—review and editing. **Carlos A. Robles‐Zazueta**: Conceptualization; data curation; formal analysis; investigation; methodology; supervision; visualization; writing—original draft; writing—review and editing. **Abelardo Montesinos‐López**: Formal analysis; investigation; methodology; writing—review and editing. **Osval A. Montesinos‐López**: Formal analysis; investigation; methodology; writing—review and editing. **Matthew P. Reynolds**: Conceptualization; funding acquisition; writing—review and editing. **Susanne Dreisigacker**: Funding acquisition; methodology; writing—review and editing. **José A. Carrillo‐Salazar**: Formal analysis; methodology. **Liana G. Acevedo‐Siaca**: Investigation; methodology; writing—review and editing. **Margarita Guerra‐Lugo**: Data curation; formal analysis; methodology. **Gilberto Thompson**: Data curation; formal analysis; methodology. **José A. Pecina‐Martínez**: Data curation; formal analysis; methodology. **José Crossa**: Conceptualization; funding acquisition; investigation; methodology; writing—review and editing.

## CONFLICT OF INTEREST STATEMENT

The authors declare no conflicts of interest.

## Supporting information




**Supplemental Table 1**: BLUEs for grain yield, grain filling percentage, grain filling rate, plant height, days to heading, stomatal conductance, transpiration and quantum yield of photosystem II in wheat.
**Supplemental Table 2**: Accuracy and NRMSE of 52 genomic prediction models using 5‐fold cross‐validation, including extended models combining different sets of variables.
**Supplemental Table 3**: Accuracy and NRMSE of 52 genomic prediction models using a Leave‐One‐Environment‐Out validation, including extended models combining different sets of variables.

**Supplemental Figure 1**: Pearson correlation of grain yield under irrigation, drought and terminal heat stress.

**Supplemental Figure 2**: Linear regressions between grain yield and transpiration, *g_s_* and ΦPSII under irrigation, drought and terminal heat stress.

**Supplemental Figure 3**: Grain yield ranges under irrigation, drought and terminal heat stress.

## Data Availability

Agronomic, physiologic, and high‐throughput traits raw and BLUEs datasets are fully available in the Dryad repository (10.5061/dryad.rbnzs7hqq). Additionally, BLUEs for agronomic and physiological traits are available as Supporting Information. For the genotypic raw dataset of this article, the data will be made available without reservations upon contacting the corresponding authors.
