# Supplementary figures and images for: Integration of physiological and remote sensing traits for improved genomic prediction of wheat yield
Source: Plant Genome. 2025 Sep 4;18(3):e70110. doi: 10.1002/tpg2.70110 (PMC12409263; doi:10.1002/tpg2.70110)

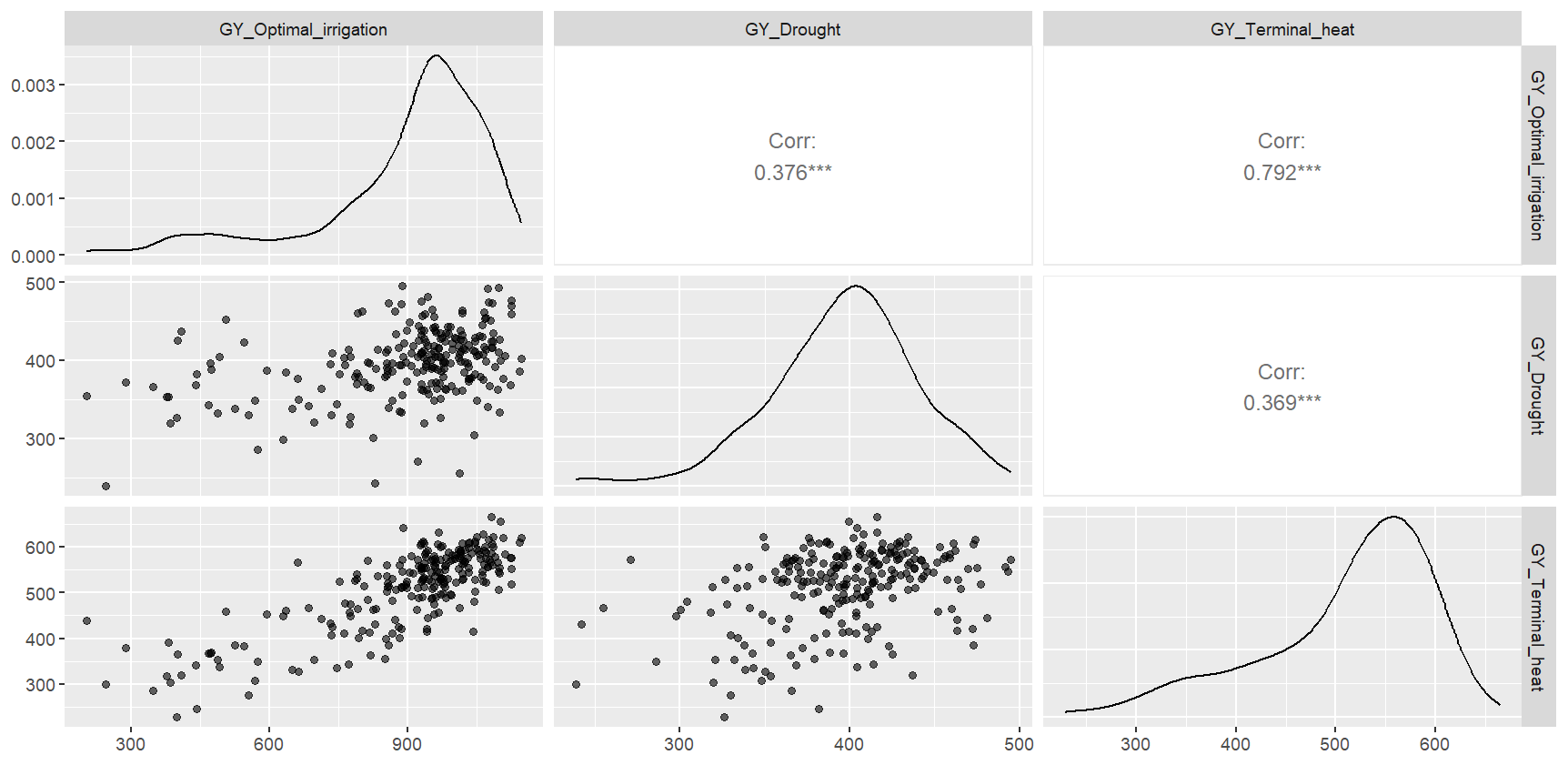

Supplement: Supplementary file 2 — Supplemental Figure 1: Pearson correlation of grain yield under irrigation, drought and terminal heat stress. [file TPG2-18-e70110-s003.tif]

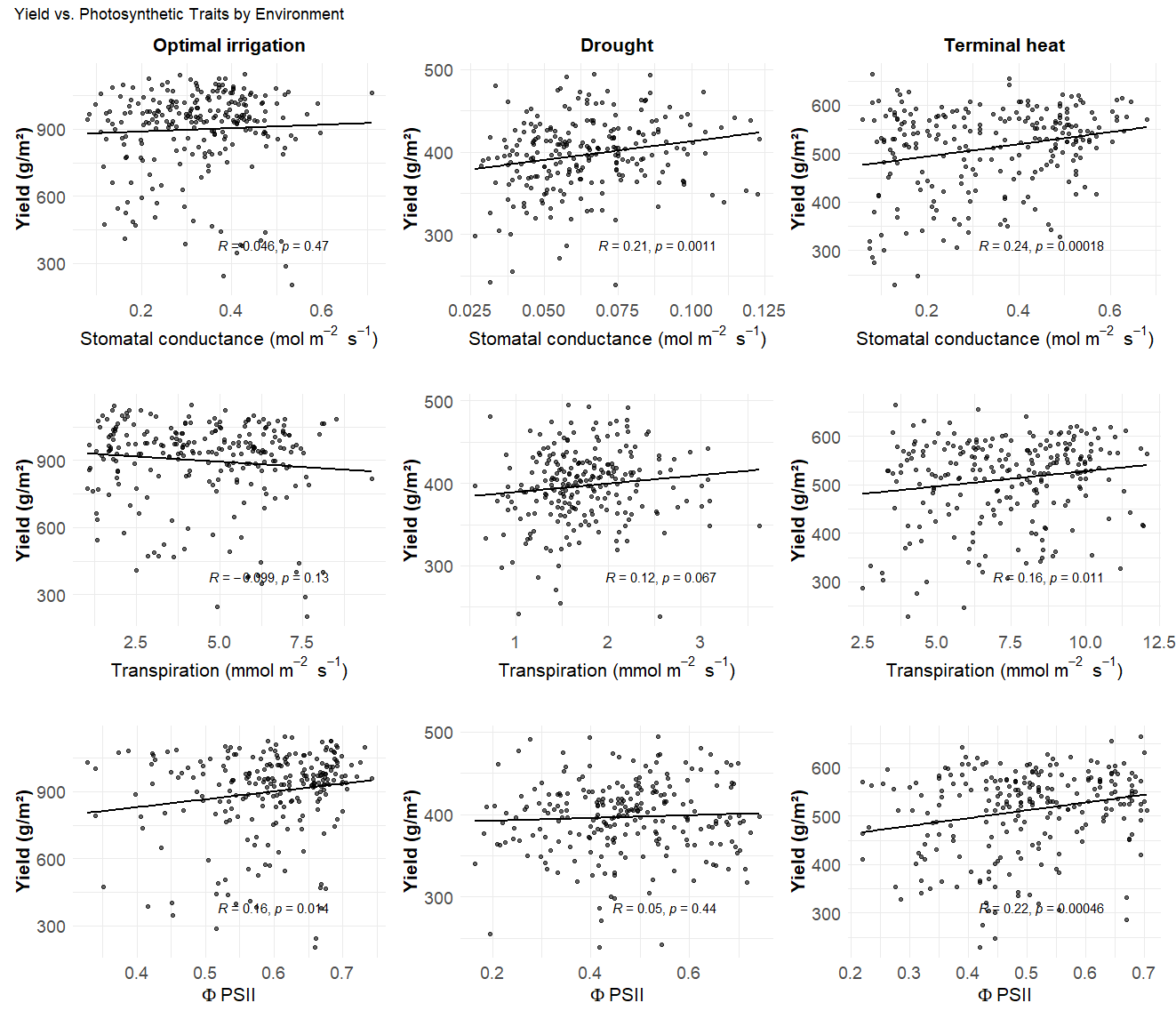

Supplement: Supplementary file 3 — Supplemental Figure 2: Linear regressions between grain yield and transpiration, gs and ΦPSII under irrigation, drought and terminal heat stress. [file TPG2-18-e70110-s004.tif]

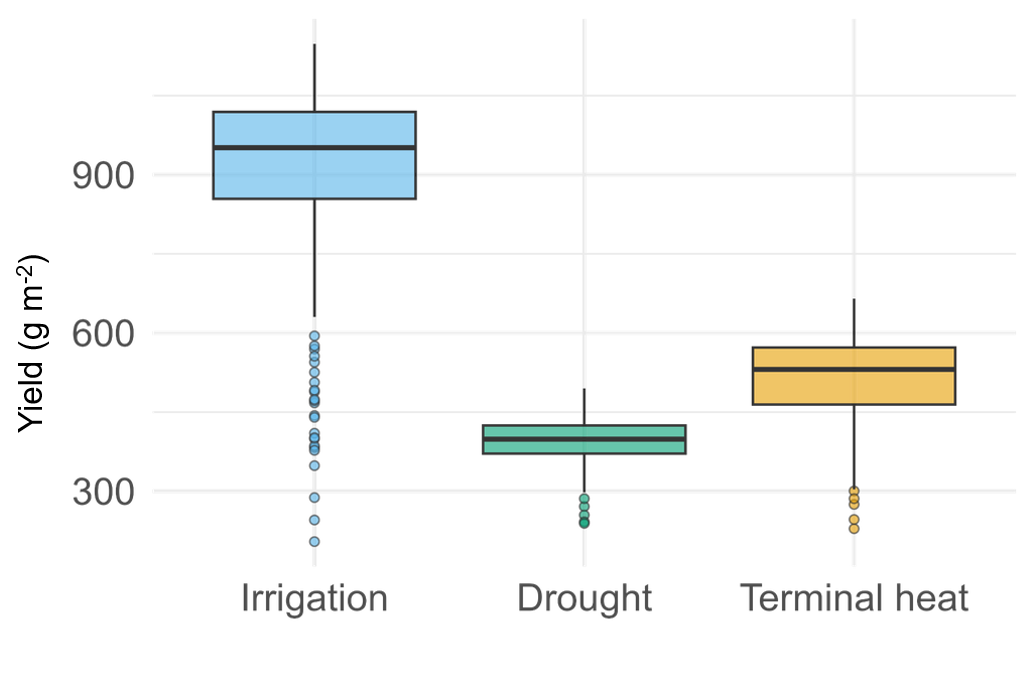

Supplement: Supplementary file 4 — Supplemental Figure 3: Grain yield ranges under irrigation, drought and terminal heat stress. [file TPG2-18-e70110-s002.tif]
